# Personality of hosts and their brood parasites

**DOI:** 10.1093/cz/zoab031

**Published:** 2021-04-02

**Authors:** Anders Pape Møller, Xingfeng Si

**Affiliations:** 1 Laboratoire d’Ecologie, Systématique et Evolution, CNRS UMR 8079, Université-Saclay, Bâtiment 362, Orsay Cedex F-91405, France; 2 Ministry of Education Key Laboratory for Biodiversity and Ecological Engineering, College of Life Sciences, Beijing Normal University, Beijing 100875, China; 3 Zhejiang Tiantong Forest Ecosystem National Observation and Research Station, School of Ecological and Environmental Sciences, East China Normal University, Shanghai 200241, China

**Keywords:** common cuckoo, competition, *Cuculus canorus*, escape behavior, host and parasite behavior, inter-specific competition, intra-specific competition, niche partitioning, tonic immobility

## Abstract

Brood parasites such as the common cuckoo *Cuculus canorus* exploit the parental abilities of their hosts, hosts avoid brood parasitism and predation by showing specific behavior such as loss of feathers, emission of fear screams and contact calls, displaying wriggle behavior to avoid hosts or potential prey, pecking at hosts and prey, and expressing tonic immobility (showing behavior like feigning death or rapid escape from predators and brood parasites). These aspects of escape behavior are consistent for individuals but also among sites, seasons, and years. Escape behavior expressed in response to a broad range of cuckoo hosts and prey are consistently used against capture by humans, but also hosts and brood parasites and predators and their prey. An interspecific comparative phylogenetic analysis of escape behavior by hosts and their brood parasites and prey and their predators revealed evidence of consistent behavior when encountering potential parasites or predators. We hypothesize that personality axes such as those ranging from fearfulness to being bold, and from neophobic to curiosity response in brood parasites constitute important components of defense against brood parasitism that reduces the overall risk of parasitism.

Predators impose strong selection pressures on their prey. Therefore, it is not surprising that a large number of different kinds of escape behavior has evolved (Curio 1987; Caro 2002). In particular, not all captured animals are dead, because a non-negligible fraction of individuals escape predation events and even escape successful capture. Such escape behavior shows diversity ranging from autotomy and feather loss (Møller et al. 2006) to fights and struggle, and feigning death, which not only have different kinds of behavior, but also their variability. Here, we suggest that escape behavior provides valuable information that scientists may use for getting a glimpse into the personality of animals, where personality constitutes consistent behavior of individuals among contexts.

Escape behavior from predators and parasites can broadly be arranged along a personality-like axis from attempts to escape to physiological changes that may allow an individual to increase the probability of escape. That would be the case if behavior would relate to anti-predation and anti-parasitism behaviors. Aggression toward a predator by prey may result in escape (Laiolo et al. 2009), including autotomy such as feather loss or loss of limbs or tails (Edmunds 1974; Vitt et al. 1977). Such behavior may allow an individual host to escape. Likewise, fear screams by prey may attract the attention of secondary predators that could interfere with the predator already holding the prey in its mouth, grip, or elsewhere (Högstedt 1983). Tonic immobility reflects the motionless state that some prey or host individuals assume upon escape, sometimes allowing an apparently lifeless individual to escape (Hoagland 1928; Jones 1986; Boissy 1995; Forkman et al. 2007). It also has similarities with studies of feigning death by prey and hosts. Elevated breath rate (Carere and van Oers 2004), heart rate (Wascher et al. 2006; Fucikova et al. 2009), and body temperature (Carere and van Oers 2004) may reflect physiological responses to escape that differ consistently among personalities and that potentially could allow an individual to escape. Finally, escape sequence estimated as the relative amount of effort that it takes to capture an individual (Birkhead et al. 1998) may reflect neophobia and hence personality differences. The plumage or pelage are used for escape, because only individuals in prime condition will be able to escape from a predator or avoid parasitism (Møller and Mateos-Gonzalez 2019). These different kinds of behavior may all and in combination have evolved as means that would facilitate escape, especially among individuals with specific personalities that are likely to escape due to their bold overall behavior (Gosling and John 1999; Sih et al. 2004; Réale et al. 2007). Such convergence in phenotype between predators and prey, but also brood parasites and hosts will select for hawk mimicry, or between predators and parasites.

The behavior of animals when escaping from a predator does not only reflect means by which specific individuals may be able to escape, but may also reflect the risks that specific categories of individuals may encounter. Such integral differences in suites of behavior that show strong covariation can be considered to reflect personalities (Wilson et al. 1994; Boissy 1995; Gosling and John 1999; Sih et al. 2004; Réale et al. 2007). Therefore, we should expect that individuals with specific personalities also experience greatly different risks of predation that should be associated with peculiar escape behavior. Individuals that show excessive escape behavior in situations with little or no risk would be selected against because their foraging activity and hence food intake is reduced. Conversely, individuals that show insufficient escape behavior would run elevated risks of remaining captured and hence enjoy no or very little probability of escape.

The objectives of this study were to quantify behavior during escape attempts or during attempts by parasites to exploit their hosts, assess the reliability of estimates of such escape behavior, and identify the potential function of escape behavior. Our working hypothesis suggested that escape behavior represents evasive behavior in a predation context because escaped individuals generally can be considered to be dead unless they somehow manage to escape. Individual animals may differ in the risk of encounters with predators, with individuals having bold and neophilic personalities being more likely to encounter such situations than cautious and neophobic individuals. If that were the case, we should expect escape behavior to reflect personality traits with specific personalities being associated with specific kinds of anti-predator and anti-parasitism behavior. Escape behavior is likely to converge between parasitic brood parasites, hawk mimicry in brood parasites, and convergence in phenotype between brood parasites and their predators (Thorogood and Davies 2013; York and Davies 2017).

## Materials and Methods

### Study sites

To analyze these questions we studied the escape behavior of birds at Kraghede (57°12′ N, 10°00′ E), Denmark (since 1971), as part of a long-term project (Møller 1994). The study site consists of open farmland with pastures, cereals, potatoes, and rape with mixed plantations, hedges, and ponds.

### Brood parasitism, number of cuckoo eggs, and cuckoo eggs

We recorded information on all 98 species of hosts of the common cuckoo. A total of 1.24% of 72 host species for which 1.24% were on average parasitized with a range from 0% to 21.89%. We recorded the prevalence of common cuckoo among host species. We also recorded the total number of cuckoo eggs in museum and private collections 1850–1940 and between 1941 and 1990.

We used intra- and inter-clutch variation in egg appearance based on data reported by Øien et al. (1995) and Soler and Møller (1996). These data are highly repeatable allowing for use of reliable data.

### Egg appearance and rejection rate

Host eggs vary within and among clutches. Hosts also vary in rejection rate among populations. We made an exhaustive compilation of data for the common cuckoo by making a literature search on ResearchGate, Google Scholar, and Web of Science.

### Escape and measurements

Birds were captured weekly during 2010–2012 from arrival in early spring until the end of the breeding season using mist nets. All recaptures were subsequently recorded, with this variable taking on values of either 0 (not recaptured again) or 1 (recaptured as least once).

Repeated measurements of the same individuals in the same season revealed that phenotypic characters had repeatabilities above 28% (Møller 1991, 1994). Hence, characters were measured in a highly consistent way. Repeatabilities for 8 characters (these are listed in Tables 1–3) ranged from *F = *1.99, *df =* 1, 97, *P = *0.012, repeatability *R = *0.28 (SE *=* 0.13) to a maximum repeatability of *F = *90.70, *df =* 1, 97, *P < *0.0001, *R = *0.97 (SE *=* 0.01).

**Table 1.** Intra-clutch variation in egg appearance in cuckoo hosts in relation to escape behavior

Residuals					
Minimum	1Q	Median	3Q	Maximum	
−0.059	0.060	−0.005	0.025	0.077	
	Estimate	SE	*t*	*P*	*R*
Intercept	0.180	0.917	0.196	0.846	
log mass	0.637	0.348	1.830	0.076	0.18
log uropygial gland	−0.378	0.274	−1.377	0.178	0.20
Biting	−0.260	0.243	−1.069	0.293	0.18
Scream	−0.518	0.424	−1.221	0.231	−0.23
Feather loss	0.117	0.330	0.353	0.712	0.14
Tonic immobility	0.007	0.011	0.593	0.557	0.089
Alarm	−0.031	0.248	−0.125	0902	0.19
Wriggle	0.165	0.206	0.802	0.428	0.23

Results from phylogenetic least squares (PGLS) models. Minimum, 1Q (first inter-quartile), median 3Q (third inter-quartile), and maximum models provide effects size (*r*) estimated as Pearson’s product-moment correlation coefficients. lambda = 0, indicating no influence of phylogeny on the regression. Residual standard error = 0.038 on 33 degrees of freedom (486 observations deleted due to missing values). Multiple *R*-squared = 0.139, adjusted *R*-squared = −0.069. *F*-statistic = 0.666 on 8 and 33 df, *P = *0.717.

**Table 2.** Inter-clutch variation in egg appearance in cuckoo hosts in relation to escape behavior

Residuals					
Minimum	1Q	Median	3Q	Maximum	
−0.126	−0.034	0.012	0.051	0.127	
	Estimate	SE	*t*	*P*	*r*
Intercept	4.441	1.708	2.600	0.014	
log mass	−0.159	0.648	−0.246	0.807	0.20
log uropygial gland	0.748	0.511	1.462	0.153	0.21
Biting	0.259	0.453	0.572	0.571	0.08
Scream	−0.074	0.791	−0.094	0.926	0.03
Feather loss	−0.701	0.615	−1.114	0.262	0.12
Tonic immobility	0.037	0.021	1.736	0.092	0.23
Alarm	−0.655	0.462	−1.417	0.166	0.18
Wriggle	−0.114	0.384	−0.298	0.768	0.20

Results from phylogenetic least squares (PGLS) models. *r* is effect size estimated as Pearson’s product-moment correlation coefficients. Minimum, 1Q (first inter-quartile), median 3Q (third inter-quartile), and maximum models provide effect size (*r*) estimated as Pearson’s product-moment correlation coefficients. lambda = 0, indicating no influence of phylogeny on the regression. Residual standard error: 0.072 on 33 degrees of freedom (486 observations deleted due to missing values). Multiple *R*-squared = 0.274, adjusted *R*-squared = 0.098. *F*-statistic = 1.555 on 8 and 33 df, *P = *0.177.

**Table 3.** Rejection rate of cuckoo eggs or egg models from nests of cuckoo hosts in relation to escape behavior

Residuals					
Minimum	1Q	Median	3Q	Maximum	
−0.111	−0.039	0.009	0.050	0.146	
	Estimate	SE	*t*	*P*	*R*
Intercept	0.280	0.878	0.319	0.752	
log mass	−0.202	0.373	−0.541	0.593	0.13
log uropygial gland	−0.020	0.189	−0.106	0.917	0.08
Biting	0.404	0.241	−1.673	0.105	0.24
Scream	−0.350	0.472	−0.742	0.464	0.16
Feather loss	0.592	0.421	1.405	0.171	0.13
Tonic immobility	0.026	0.012	2.275	0.031	0.26
Alarm	−0.284	0.224	−1.264	0.217	0.21
Wriggle	0.002	0.234	0.010	0.992	0.04

Results from PGLS models provide effect size (*r*) estimated as Pearson’s product-moment correlation coefficients. lambda = 0.988, indicating a strong influence of phylogeny. Residual standard error: 0.067 on 28 degrees of freedom (491 observations deleted due to missing values). Multiple *R*-squared = 0.291, adjusted *R*-squared = 0.088. *F*-statistic: 1.435 on 8 and 28 df, *P = *0.226.

We used information on the size of the uropygial gland as an estimate of the ability of hosts to provide secretions from the gland to the plumage and hence the ability to maintain a functional plumage. We hypothesize that a large uropygial gland that produces ample amounts of secretions will allow for efficient escape from brood parasites (Møller et al. 2010). Such secretions may provide defense by cuckoo hosts against common cuckoos (Møller and Mateos-Gonzalez 2019), increased fecundity with higher amounts of secretions (Magallanes et al. 2017), and reduced risk of malarial infection and higher survival rate (Magallanes et al. 2019).

### Escape behavior

We quantified 7 aspects of behavior during and immediately following escape. (1) Wriggle score. The measure quantifies how much a bird budges while held in a hand (with a score of 0—no movement, 1—moves rarely, 2—moves regularly, but not always, and 3—moves continuously). (2) Biting. Holding an index finger in front of the beak and giving a score of 0, if the bird does not peck, or 1 if it does. (3) Breath rate. The number of inhalations recorded during 30 s with the fingers on the breast muscles. (4) Escape sequence. The relative ranking from 0 to 1 of the sequence of all escapes in a given year. (5) Fear scream. Whether the bird gives a fear scream when held in the hand. (6) Tonic immobility. Just before a bird is released, we placed it with my right hand on its back on my flat left hand. When the bird was lying still, we removed the right hand and recorded time until the individual righted itself and flew away. If the bird had not flown away after 30 s, we terminated the trial. (7) Alarm call. Whether the bird gave an alarm call when departing from your hand. (8) Feather loss. Whether the individual lost feathers when removed from the mist net or while held during measurements and scoring of behavior.

### Statistical analyses

We used an effect size approach to describe the relationships between escape behavior and aspects of brood parasitism. Rather than using significance approaches to evaluate statistical results (Perneger 1998; Garamszegi 2006), we here use an effect size approach (Holm 1979; Wright 1992; Chandler 1995; Perneger 1998; Garamszegi 2006). We calculated effect sizes in terms of Pearson’s *r*. We adopted the guidelines of Cohen (1988) as a yardstick, suggesting that *r *=* *0.10 explaining 1% of the variance is a small effect, *r *=* *0.30 explaining 9% of the variance is an intermediate effect, and *r *=* *0.50 explaining 25% of the variance is a large effect. We estimated effect size as Pearson’s product-moment correlation coefficients, using the equations in Rosenthal (1991), Cooper and Hedges (1994), and Hedges and Olkin (1985). A review of all published meta-analyses in biology revealed that the average effect size was approximately *r *=* *0.22–0.26 explaining 5–7% of the variance. We weighted statistical tests by sample size to weight results. Such weighting can be more important than adjustment of phylogenetic analyses by similarity due to common phylogenetic descent (Garamszegi and Møller 2010).

### Phylogenetic comparative analyses

We made multivariate phylogenetic generalized least squares (PGLS) models for the comparative analyses using the function “pgls” in R package “caper” (Orme et al. 2018). Phylogenetic dependence, as estimated using Pagel’s *λ*, was set to the most appropriate value assessed by maximum likelihood in each model. We downloaded 1,000 pseudo-posterior phylogenetic trees from birdtree.org published by Jetz et al. (2012) under the option “Hackett All Species: a set of 10,000 trees with 9993 OTUs each”. We constructed a maximum clade credibility tree with mean node heights using TreeAnnonator v1.8.2 in the BEAST package (Drummond and Rambaut 2007) for following phylogenetic comparative analyses.

## Results

### Rates of parasitism

We found 98 species of hosts with information on escape behavior, and the parasitism rate was on average 1.24% among 72 species of potential hosts ranged from 0% to 21.89% of nests were parasitized. Parasitism rate was positively related to the log_10_ number of cuckoo eggs (*F = *13.67, *df =* 1, 60, *P < *0005, estimate [SE] = 0.015 [0.004]) until 26 September 2014. Parasitism rate was positively related to the log_10_ number of cuckoo eggs in museum collections across the globe 1850–1940 (*F = *9.99, *df =* 1, 60, *P = *0.0025, estimate [SE] = 0.015 [0.004]). There was also a positive relationship between the number of cuckoo eggs during 1941–1990 expressed as the log_10_ number of cuckoo eggs (*F = *19.34, *df =* 1, 60, *P < *0.0001, estimate [SE] = 0.017 [0.004]). There was also a positive relationship for the log_10_ number of cuckoo eggs recorded during 1990–2013 (Saino et al. 2009; Møller et al. 2011; *F = *177.40, *df =* 1, 66, *P < *0.0001, estimate [SE] = 0.352 [0.026]). We assumed that the first estimate was an estimate of the number of cuckoo eggs in the following analyses.

### Intra-clutch variation in egg appearance and escape behavior

PGLS models showed an intermediate effect of −0.23 for fear screams implying that fear screams were less common at higher levels of intra-clutch variation (Table 1). There was also a small effect of +0.23 for wriggling implying that species that wriggled more had higher intra-clutch variation (Table 1).

### Inter-clutch variation in egg appearance and escape behavior

A PGLS showed an intermediate effect of tonic immobility of +0.23 suggesting that species with greater inter-clutch variation also had higher level of tonic immobility (Table 2). There was also an intermediate effect of +0.21 suggesting that the size of the uropygial gland was larger in host species with greater intra-clutch variation in egg appearance.

### Rejection rate of egg appearance and escape behavior

A PGLS revealed that rejection rate was larger in species with higher level of tonic immobility (Table 3). This intermediate effect of +0.26 suggested that tonic immobility lasted longer in host species with higher rate of egg rejection.

## Discussion

Personality has in recent years been related to many aspects of animal behavior. It is only recently that personality has been related to brood parasitism (Aviles and Parejo 2011; Campobello and Sealy 2011). For example, the hormonal background has proven to be important for brood parasitism (Abolins-Abols and Hauber 2018). Furthermore, age and experience may affect the probability of brood parasitism and hence the diverse reactions of potential hosts to brood parasites (Lotem et al. 1995). Here, we have shown that hosts of brood parasites behave in specific ways when captured for identification, measurement, and sampling. Specifically such escape behavior that can be considered to represent anti-predator behavior (Møller et al. 2011) is related to inter- and intra-clutch variation in egg appearance and rejection rate of cuckoo eggs. Escape behavior ranges from struggling to escape, aggression directed toward the capturer, screams that may resemble death screams, and loss of feathers when brood parasites physically interact with potential hosts to physiological responses such as increased breath rate, and tonic immobility, and to escape behavior such as escape flight and alarm calls when escaping. Tonic immobility in hosts is the duration that hosts spend immobile when encountering a possible brood parasite. Therefore, tonic immobility is an important component of fitness (Møller and Ibáñez-Álamo 2012) that is also related to domestication (Jones 1986; Boissy 1995; Forkman et al. 2007; Campler et al. 2009). Such escape behavior is highly consistent among capture events, but also among individuals of the same species. Previous studies of escape behavior were related to body mass, mating success, fecundity, and probability of local recruitment (Møller and Ibáñez-Álamo 2012). Here, we reported effect size estimated as Pearsons’s product-moment correlation coefficients. Effect size is an estimate of the strength of the relationship between 2 or more associations. Most of the effect sizes for escape behavior that we reported here were small to intermediate accounting for 1–10% of the variance, as is commonly the case in biology. However, we emphasize that among the 21 effect sizes reported in Tables 1–3, there were 12 that were significant at the 5% level, which is more than expectation (12.0 vs. 0.05), Repeatability of escape behavior was consistent among capture events in different species of birds with some characters showing a high degree of viability while others were consistent (Møller and Ibáñez-Álamo 2012). There was little evidence of significant correlations among different kinds of escape behavior implying that these generally reflected statistically independent measures (Møller and Ibáñez-Álamo 2012). Here, we have shown that domestic animals show restraint and capture behavior that differs from that of their wild counterparts. For example, domestic animals show little or no fear reactions and no stress responses when approached by humans or domestic animals such as dogs (e.g. Kohane and Parsons 1988; Geffroy et al. 2020). Such behavioral adaptation to domestication has been experimentally induced in fruitflies (Kohane and Parsons 1986, 1987), foxes (Belyaev 1969, 1979; Trut et al. 2009), and chickens (Campler et al. 2009; Wirén et al. 2009). Escape behavior has a genetic basis and responds to artificial selection, as shown by rapid change in escape behavior during the domestication process (Geffroy et al. 2020).

Life history is central to studies of personality because life history trade-offs constitute one reason why no single personality prevails in heterogeneous environments. Some personalities do better in one environment, while others do well elsewhere, and none do well everywhere (McElreath et al. 2007; Stamps 2007; Wolf et al. 2007; Biro and Stamps 2008; Garamszegi et al. 2008, 2009; Careau et al. 2009). Here, we have shown that aspects of escape behavior are significantly correlated with components of fitness as reflected by inter- and intra-clutch variation in egg appearance among host eggs. In addition, escape behavior was related to rejection rate of common cuckoo eggs. This suggests that tonic immobility at least is phenotypically correlated with important components of fitness associated with intra- and inter-clutch variation in egg appearance and rejection rate of cuckoo eggs.

In conclusion, we have shown that different aspects of behavior displayed by host birds during escape and subsequent handling as reflected by tonic immobility were related to intra- and inter-clutch variation in egg appearance with small to intermediate effect sizes and rejection rate of cuckoo eggs.
